# Connections between Diabetes Mellitus and Metabolic Syndrome and the Outcome of Cardiac Dysfunctions Diagnosed during the Recovery from COVID-19 in Patients without a Previous History of Cardiovascular Diseases

**DOI:** 10.3390/biology12030370

**Published:** 2023-02-26

**Authors:** Cristina Tudoran, Renata Bende, Felix Bende, Catalina Giurgi-Oncu, Alexandra Enache, Raluca Dumache, Mariana Tudoran

**Affiliations:** 1 Department VII, Internal Medicine II, Discipline of Cardiology, University of Medicine and Pharmacy “Victor Babes” Timisoara, E. Murgu Square, Nr. 2, 300041 Timisoara, Romania; 2 Center of Molecular Research in Nephrology and Vascular Disease, Faculty of Medicine, University of Medicine and Pharmacy “Victor Babes” Timisoara, E. Murgu Square, Nr. 2, 300041 Timisoara, Romania; 3 County Emergency Hospital “Pius Brinzeu”, L. Rebreanu, Nr. 156, 300723 Timisoara, Romania; 4 Academy of Romanian Scientists, Ilfov Str., Nr. 3, 50085 Bucuresti, Romania; 5 Center of Advanced Research in Gastroenterology and Hepatology, Faculty of Medicine, University of Medicine and Pharmacy “Victor Babes” Timisoara, 300041 Timisoara, Romania; 6 Department VII, Internal Medicine II, Discipline of Gastroenterology, University of Medicine and Pharmacy “Victor Babes” Timisoara, E. Murgu Square, Nr. 2, 300041 Timisoara, Romania; 7 Department VIII, Neuroscience, Discipline of Psychiatry, University of Medicine and Pharmacy “Victor Babes” Timisoara, E. Murgu Square, Nr. 2, 300041 Timisoara, Romania; 8 Department VIII, Discipline of Forensic Medicine, University of Medicine and Pharmacy “Victor Babes” Timisoara, E. Murgu Square, Nr. 2, 300041 Timisoara, Romania; 9 Center for Ethics in Human Genetic Identification, University of Medicine and Pharmacy “Victor Babes” Timisoara, E. Murgu Square, Nr. 2, 300041 Timisoara, Romania

**Keywords:** COVID-19, diabetes mellitus type 2, insulin resistance syndrome, obesity, metabolic syndrome, inflammation, transthoracic echocardiography, diastolic dysfunction

## Abstract

**Simple Summary:**

In this original article, we aimed to describe the immense influence of an augmented metabolic risk profile, such as the case of type 2 diabetes mellitus, metabolic syndrome, and obesity, on the evolution of a SARS-CoV-2 virus infection, with a focus on the cardiovascular abnormalities encountered in post-acute COVID-19 syndrome. We demonstrated that during the recovery from COVID-19, the above-mentioned pathologies, associated with an increased inflammatory burden, favor the development of various cardiac alterations—which are diagnosable by transthoracic echocardiography—in previously healthy individuals. At the 3- and 6-month follow-up, we observed that the echocardiographic parameters characterizing the left and right ventricular function, as well as the increased pressure in the pulmonary artery, had improved, which was not the case for diastolic dysfunction (mostly of type 3). These cardiac pathologies, such as the altered systolic and diastolic functions and/or the presence of pulmonary hypertension, could explain—at least partially—the development of long COVID-19 syndrome. Therefore, besides the usual post-COVID-19 assessments, patients with an increased metabolic risk profile should be supplementarily evaluated by a cardiologist, including by a comprehensive echocardiography, both during the acute infection as well as in the recovery period.

**Abstract:**

(1) Background: Throughout the COVID-19 pandemic, it became obvious that individuals suffering with obesity, diabetes mellitus (T2DM), and metabolic syndrome (MS) frequently developed persisting cardiovascular complications, which were partially able to explain the onset of the long-COVID-19 syndrome. (2) Methods: Our aim was to document, by transthoracic echocardiography (TTE), the presence of cardiac alterations in 112 patients suffering from post-acute COVID-19 syndrome and T2DM, MS, and/or obesity, in comparison to 91 individuals without metabolic dysfunctions (MD); (3) Results: in patients with MD, TTE borderline/abnormal left (LVF) and/or right ventricular function (RVF), alongside diastolic dysfunction (DD), were more frequently evidenced, when compared to controls (*p* ˂ 0.001). Statistically significant associations between TTE parameters and the number of factors defining MS, the triglyceride-glucose (TyG) index, the severity of the SARS-CoV-2 infection, and the number of persisting symptoms (*p* ˂ 0.001) were noted. Significant predictive values for the initial C-reactive protein and TyG index levels, both for the initial and the 6-month follow-up levels of these TTE abnormalities (*p* ˂ 0.001), were highlighted by means of a multivariate regression analysis. (4) Conclusions: in diabetic patients with MS and/or obesity with comorbid post-acute COVID-19 syndrome, a comprehensive TTE delineates various cardiovascular alterations, when compared with controls. After 6 months, LVF and RVF appeared to normalize, however, the DD—although somewhat improved—did persist in approximately a quarter of patients with MD, possibly due to chronic myocardial changes.

## 1. Introduction

Ever since the beginning of 2020, as the infection with a new variant of the severe acute respiratory syndrome (SARS-CoV-2) virus spread worldwide and progressed rapidly to an alarming pandemic, it was evident that the severity, prognosis, and mortality rates of COVID-19 varied largely among infected populations [1,2,3]. Surprisingly, a worse evolution, with a large spectrum of systemic complications—often requiring admission in intensive care units (ICUs) and resulting in fatal outcome—was observed, not only in elderly and frail patients that had multiple comorbidities, but also in younger, apparently healthy subjects, especially when they had associated metabolic dysfunctions such as visceral obesity, metabolic syndrome (MS), and type 2 diabetes mellitus (T2DM) [4,5,6]. 

**Figure 1:**
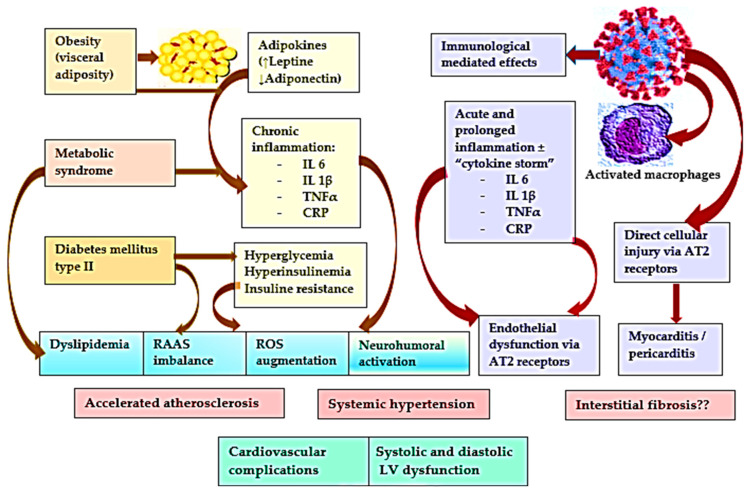
Connections between the pathophysiological processes involved in metabolic dysfunctions and in COVID-19. Legend: IL—interleukin; TNF—tumor necrosis factor; CRP—C reactive protein, AT—angiotensin; RAAS—renin-angiotensin-aldosterone system; ROS—reactive oxygen species.

Currently, it has been proven that theSARS-CoV-2 virus exerts its actions both directly, by binding on the cell surface receptors, but also through immunological mediated effects, by activating the innate and adaptive immunity. Therefore, the virus determines the release of large amounts of proinflammatory cytokines, namely interleukine-6 (IL-6) and interleukine-1β (IL-1β), but also of other acute phase mediators, such as ferritin and C-reactive protein, Figure 1. In some individuals, these immune responses can become exaggerated, resulting in an augmented release of cytokines—namely, the “cytokine storm” [7]. Another potential pathway is mediated via the macrophages, responsible for the initiation of the hypoxia-inducible factor (HIF-1α) [7,8]. Although the lungs represent the first target, it has often been suggested that cardiovascular implications are frequent in COVID-19, both in its acute stages as well as during recovery [9,10]. Initial myocardial damages, such as myocarditis, heart failure, or even necrosis, can be explained by the direct action of the virus on the myocytes and vessels, and the subsequent inflammation, endothelial dysfunction, and ischemia [11]. These pathophysiological processes may persist even during the recovery phase, explaining—at least partially—the development of an interstitial fibrosis, resulting in myocardial stiffening with left ventricular (LV) changes, and determining alterations in cardiac contraction and relaxation—frequently associated with the occurrence of heart failure, with a reduced (HFrEF), or preserved ejection fraction (HFpEF) [11,12], Figure 1. A preexisting enhanced pro-inflammatory risk profile, characterized by metabolic imbalance and augmented inflammatory processes—associated with high levels of IL-6 and IL-1β cytokines, alongside adipose tissue-derived TNFα and leptin in subjects with obesity, MS, and T2DM—contributes to an exacerbated immunologic response of “hyper-inflammation” during COVID-19, with deleterious effects [5,6,13], Figure 1. It has been proven that insulin resistance (IR), favored by elevated cytokine levels, represents the hallmark of T2DM, often long preceding its occurrence, but also characterizing MS and obesity [14,15,16]. In obesity, there is an augmented activity of the IL-6 receptor, which, despite elevated levels of circulating cytokines, results in an exacerbated inflammatory state—namely, the so-called meta-inflammation. Moreover, in this metabolic pathology, IR favors the infiltration of the adipose tissue with macrophages, particularly of the highly inflammatory type M1 subpopulation [17,18]. Therefore, lipotoxicity- and glucotoxicity-modulated IR tends to amplify the cardiovascular risk in patients with metabolic dysfunctions, thus favoring the development of systemic hypertension, and left ventricular hypertrophy, frequently associated with DD [19,20]. It, therefore, becomes easy to assume that, in the situation of an already exacerbated inflammatory background, an additional inflammatory burden, such as COVID-19, would trigger multiple systemic injuries, with a worse evolution [21,22,23]. Additionally, as already debated in the medical literature, in some patients recovering from this infection—especially when they have also been impacted by comorbid T2DM, MD, and obesity—the restoration of normal immunologic responses becomes deficient or/and delayed, further resulting in an immunologic depression [17,24,25]. These augmented immune responses may persist during the recovery phase, and, in some cases, a reactivation of the viral infection has been described via mechanisms that, in aggregate, could be responsible for the development of post-COVID syndrome, which is characterized by a persistence of a large spectrum of symptoms [26,27]. As expected, specifically cardiovascular complications tend to occur more frequently and have a worse evolution in individuals with an unfavorable metabolic risk profile, even when there is no pre-existing history of cardiac diseases or other health issues [22,26]. Even in patients who are free of any identifiable cardiovascular complications during the acute phase of COVID-19, subtle abnormalities may be diagnosed by means of TTE, which seem to persist long into the recovery [28,29]. 

Our objective was to document the presence of any cardiac alterations assessed by TTE in post-acute COVID-19 syndrome patients during their recovery from a mild/moderate infection. These individuals were identified to have an increased cardio-metabolic risk profile due to metabolic dysfunctions, but undiagnosed with cardiovascular diseases. Another aim was to highlight the potential connections between the severity and evolution of these TTE abnormalities, and several clinical and laboratory parameters characterizing the amplitude of the metabolic imbalance and COVID-19 consequences as well.

## 2. Materials and Methods

### 2.1. Study Population 

We evaluated 203 patients suffering with post-acute COVID-19 syndrome between March 2021 and March 2022 in the ambulatory services of our hospital. A longitudinal study was conducted; patients who met the study’s inclusion criteria were identified and subsequently followed for a period of 6 months from the first evaluation. The initial evaluation was performed using clinical criteria, laboratory tests, ECG, and TTE, and afterward, all subjects were reevaluated clinically and by TTE at 3 and 6 months, respectively. The patient sample was selected from a larger population of 483 COVID-19 convalescents who attended our medical services for various non-specific complaints, with the most frequent ones being reduced physical exertion capacity, persisting fatigue, palpitations, elevated blood pressure levels, chest discomfort or even pain, dyspnea, dry cough, sleep disturbances, foggy brain, and concentration issues. After a detailed clinical examination of all patients, they were diagnosed as having post-acute COVID-19 syndrome, based on the persistence of symptoms for more than 4 weeks after the onset of the acute infection, but for less than 3 months. Those found with various physical sequelae and/or significant abnormalities were referred for further investigations and appropriate interventions. Of the remaining subjects, we identified 283 individuals younger than 55 years who reported an adequate health status before contracting the SARS-CoV-2 infection, without any history or signs suggesting preclinical or clinical cardiovascular diseases, nor previous therapies for chronic diseases, but with an inappropriate cardiometabolic risk profile [30,31,32,33,34,35]. We offered them the opportunity to take part in our study and to undergo further medical examinations, including lab tests, electrocardiogram (ECG), and TTE, once we ensured they fulfilled our inclusion/exclusion criteria and accepted to sign an informed consent form. Patients were requested to provide a discharge letter or an ambulatory evaluation certifying this infection, confirmed by a positive result of real-time reverse transcriptase–polymerase chain reaction (RT-PCR) testing of pharyngeal and nasal swabs, with additional lab tests, pulmonary radiography or thoracic CT scans, ECG evaluation, as well as recent medical documents, containing a physical exam, ECG and TTE results (even in an abbreviated form), and laboratory data (lipid panel, fasting blood glucose and uric acid levels), to attest their previous health status. We required these results in order to confirm or exclude a baseline metabolic dysfunction.

A subsample of 238 subjects agreed to take part in our study and were able to provide the necessary documents, while also fulfilling the inclusion criteria, which were as follows: (a) apparently healthy subjects, in the age range of 18–55 years, who were able to understand and sign the informed consent form; (b) the evidence of a recent mild/moderate SARS-CoV-2 infection, certified by a positive result of a RT-PCR assay with a baseline medical assessment, including laboratory blood tests, ECG, and chest radiography or CT scan, which allowed us to classify the severity based on the extent of the lung injury, as follows: 0–15% defining mild, and 15–40% moderate forms; (c) the availability of a recent medical evaluation (of less than one year) indicating a lack of chronic health issues, significant cardiovascular diseases, or therapies for various metabolic illnesses, even if they were occasionally found with elevated BBG, abnormal lipids panel, or if they were obese or overweight, meeting the criteria for MS.

The exclusion criteria consisted of the following: (a) subjects not able or willing to sign the informed consent; (b) individuals older than 55 years, with an increased probability to have a significant underlying cardiovascular condition; (c) those recovering from a severe form of COVID-19 illness, with certified cardiovascular complications during the acute illness or those with asymptomatic forms or without a medical evaluation during the infection; (d) patients previously confirmed with cardiovascular diseases or being treated for a chronic disease or diagnosed during the initial assessment with a significant cardiac dysfunction; (e) subjects already registered and managed for T2DM; (f) a lack of recent medical assessments.

### 2.2. Study Procedures and Clinical and Laboratory Examinations

- Patient evaluation: for the 238 participants, after they signed the individual informed consents, we gathered all available medical information regarding the course of the acute phase of the infection, along with their most recent health assessments. First, we evaluated the gravity and consequences of the infection from medical records containing results of the chest radiography or CT describing the extent of the lung injury, an ECG, and blood tests. Subsequently, we focused on the analysis of the pre-COVID-19 health status and several clinical and laboratory parameters of interest for this study, such as the presence of chronic diseases (those with current therapies for systemic hypertension or T2DM were excluded from our study [36,37,38,39,40,41], but occasionally elevated or borderline values of blood pressure or BBG were accepted), mentions regarding body weight and height, health risk, blood pressure values, and ECG and TTE results (even if considered as normal). Subsequently, all patients had an ECG and TTE to identify any significant cardiovascular alterations that could have been missed in the previous evaluations. We repeated these examinations at 3 and 6 months for all study patients.
- Echocardiographic examination: Every TTE determination was performed following guideline recommendations. After the standard measurements of all cardiac structures and the assessment of their function, from a long axis parasternal view, we determined the left ventricular (LV) mass index (LVMI), LV hypertrophy (LVH), and confirmed by LVMI over a value of 115 g/m^2^ (men) or 95 g/m^2^ (women). The following parameters, characterizing four patterns of cardiac abnormalities, were also evaluated.

(a) The borderline LV function (LVF) was appreciated from an apical 2-, 3-, and 4-chamber view by determining the following:

− The Left Ventricular ejection fraction (LVEF), calculated according to the Simpson method (modified) formula (results under 50% considered as pathological).
− The MAPSE (lateral mitral annular plane systolic excursion), with values lower than 10 mm appreciated as pathological.
− We assessed the Left Ventricular global longitudinal strain (LV-GLS) by speckle tracking, and automatically generated the ROI (region of interest) after tracing the Left Ventricular endocardial border, with manual adjustments as needed, in order to adjust the width of the LV wall [8,19]. An impaired LVF was represented by values lower than −18%, while scores between −18 and −19 were considered as borderline values [9,11].

(b) The right ventricular function (RVF) and estimated systolic pulmonary artery pressures (sPAP) were also assessed from an apical 4-chamber view:

− We measured the TAPSE (tricuspid annular plane systolic excursion), in M-Mode, at the lateral tricuspid valve annulus level, and considered values below 17 mm as abnormal.
− From an apical view, we determined the FAC (fractional area change), and deemed any scores lower than 35% as representative for a Right Ventricle dysfunction (RVD).
− By using strain techniques and employing the same view, the RV global longitudinal strain was assessed; values lower than −28% certified an RVD [28,29].
− We determined the sPAP by looking at the velocity of the peak tricuspid regurgitation (TRV) assessed by a continuous Doppler, while considering the pressure in the right atrium (RAP), appreciated in terms of the diameter of the inferior vena cava (IVC) as well as its respiratory differences. For this study, any resting sPAP values above 35 mm Hg were suggestive of a PH [36], with severities in the mild (35–44 mmHg) to either a moderate (45–60 mmHg) or severe range (above 60 mmHg).

(c) The Left Ventricular diastolic dysfunction (DD) was measured with the following parameters:

− The volume index of the left atrium (LAVI) was measured from an apical 4-chamber view, with scores above 34 mL considered pathological.
− At the mitral valve level, we used a pulsed Doppler with a similar interpretation for recording mitral inflow and measuring the early peak diastolic velocity (E), as well as the late diastolic velocity (A); subsequently, an E/A ratio was calculated.
− We used tissue Doppler imaging (TDI) at the septal and lateral mitral annulus levels to measure early (e’) and late diastolic velocity (a’); average and E/e’ ratios were subsequently calculated.

Type I DD was indicated by an E/A ratio ≤ 0.8 and E ˂ 50 cm/s; a type III DD consisted of an E/A ratio above 2. Any E/A ratios lower than 0.8, alongside E values above 50 cm/s, or E/A scores of between 0.8 and 2, suggested a type II DD by at least two of the following three criteria: average E/e’ values above 14, LAVI over 34 mL/m^2^, and/or a TRV above 2.8 m/s. A type I DD was indicated when only one of the above criteria were present [11,42].

(d) We used standard views to assess the presence/amount of the pericardial exudation (PE), and/or of the width of the posterior pericardium (PT) [29].

- Physical and laboratory examination: Because of these assessments, a further 35 patients had to be excluded from our study due to previously unidentified significant health conditions. We measured the BMI (≥30 kg/m^2^ indicating obesity) and waist circumference (WC) for the remaining 203 subjects, followed by blood sample collection to determine the BBG, serum creatinine, and the calculation of eGFR, uric acid, total (TChol), low-density lipoprotein (LDLChol), and high-density lipoprotein (HDLChol) cholesterol levels, triglyceride (TG), and C-reactive protein (CRP). T2DM was certified by a BBG exceeding 126 mg/dL twice in non-consecutive days, and a glycated hemoglobin exceeding 6.5%; MS was defined by the presence of three or more of the following factors: IMC ≥ 30 kg/m^2^, WC ≥ 102 cm in men and ≥88 cm in women, impaired glucose metabolism, HDLC ˂ 40 mg/dL in men and ˂50 mg/dL in women, TG ≥ 150 mg/dL, and uric acid ˃ 6.5 mg/dL, and BP ˃ 135/85 mmHg. Several significant indexes for the evaluation of MS were calculated, as follows:

− The triglyceride-glucose index (TyG) represents the logarithm of the product of BBG and fasting TG, the formula being: ln[BBG(mg/dl) × TG (mg/dl)/2]. This has been recommended as an alternative indicator for IR because it correlates to lipotoxicity and glucotoxicity [35,43]. A close relationship has been demonstrated between the TyG and cardio metabolic outcomes, T2DM, endothelial dysfunction, systemic hypertension, cardiovascular diseases, stroke, and—more recently—with patient outcome in COVID-19 [34,35,44]. In the medical literature, the normal cut-off values reported for TyG vary widely between 4 and 8 (due to the position of 2 in the TyG index formula) [34,35].
− The product of lipid accumulation (LAP), which is accepted as an indicator for visceral adiposity, was calculated based on WC and fasting TG. The formulas are: LAP = (WC(cm) − 65) × TG(moll⁄) (men); LAP = (WC(cm) − 58) × TG(moll⁄) (women). Reference LAP cut-off values range between25.16 to 31.59 cm × moll/l (women), and between 20.10 and 63.89 cm × moll/l (men). LAP is largely employed as an indicator for MS, abdominal obesity, and is deemed as a health risk for cardiovascular illnesses, predicting adverse cardiovascular events [45,46].
− We calculated the VAI (visceral adiposity index) with the following formulas: VAI = (WC(cm)/(39.68 + (1.88 × BMI) × (TG/1.03) × (1.31/HDL) (men) and VAI = (WC(cm)/(36.58 + (BMI × 1.89) × (TG/0.81) × (1.52/HDL) (women).

To quantify the physical consequences of the SARS-CoV-2 infection based on the number of persisting symptoms and to evaluate the rehabilitation process, we used the Post-COVID-19 Functional Status (PCFS) assessment scale. This methodology was created to quantify the amplitude of functional limitations. Based on this assessment tool, the absence of symptoms/limitations is quoted as 0; discreet limitations of quotidian activities associated with few symptoms represents a 1; a slight limitation, but with more significant symptoms is scored as a 2; a moderate limitation, associated with the inability to perform usual activities due to persistent symptoms, but still capable to take care of themselves without someone’s help, is scored as a 3; a severe physical limitation due to severe symptoms requiring support for taking care of themselves is scored as a 4 [46]. 

### 2.3. Statistical Analysis

We used the MedCalc Version 19.4 (MedCalc Software Corp., Brunswick, ME, USA) and Microsoft Office Excel 2019 (Microsoft for Windows) for the statistical analysis, while for the demographic, anthropometric, and clinical data, patients’ descriptive statistics were used. The sample size determination was performed using α as the selected level of significance and Z 1-α/2 as the value from the standard normal distribution holding 1-α/2 below it. We used the following parameters: α = 0.05, therefore 1-α/2 is 0.975 and Z is 1.960. Using these parameters, a sample size of 134 or more subjects was defined as statistically significant. The distribution of numerical variables was tested using the Kolmogorov–Smirnov test and continuous numerical variables with normal distributions were presented as means with standard deviations (SDs); in case of variables with non-normal distributions, we employed median and interquartile ranges (IQRs); the categorical variables were communicated as frequencies and percentages. The Student’s t-test was utilized for group comparisons of continuous variables with normal distribution; for variables with non-normal distribution, we applied a Mann–Whitney U-test. We employed Pearson’s χ2-test for categorical variables group comparisons. We used Spearman’s correlation test to gauge the associations between the LV-, and RV-GLS, as well as the E/e’, PT (pericardial thickness), and several other demographic, anthropometric, laboratory, and echocardiographic findings. We deemed a *p*-value lower than 0.05 as significant for all statistical analyses.

The uni-and multivariate regression analyses were used for the classification of any objective predictors of an occurrence of cardiac deviations. Three multivariate regression models were built using the Akaike criterion to assess the impact of several factors on the variance of continuous variables, and the model was validated based on the accuracy of prediction and R squared. In the final regression equations, the predictors were accepted according to a repeated backward-stepwise algorithm (inclusion criteria *p* < 0.05, exclusion criteria *p* > 0.10) to obtain the most appropriate theoretical model to fit the collected data.

The Local Scientific Research Ethics Committee of the hospital approved our study (206/07.2020 and 297/11.04.2022).

## 3. Results

Our study was conducted on 203 patients, with ages ranging from 26–55 years old, and a mean age of 47.06 ± 7.65 years, including 82 men (40.39%) and 121 women (59.60%), all of whom were diagnosed with a SARS-CoV-2 virus infection 63 [56–70] days prior to attending this medical evaluation. In terms of any identifiable metabolic dysfunctions, they were allocated to three subgroups: group I included 46 patients with DM and MS, group II had 66 subjects with MS, while group III consisted of 91 individuals with normal weight or who were overweight, but without clinical MS or obesity. All the patients’ clinical characteristics are presented in Table 1, and all laboratory data are available in Table 2. TTE parameters, as analyzed during the first evaluation, can be seen in Table 3. 

Group I included 46 patients (16 men and 30 women), with an average age of 52 ± 3.54 years old; most of them had elevated BMI, with a median value of 30.96 [29.22–32.86] kg/m^2^, while 29 were estimated to pertain to the obesity category (24 of them in the 1st degree, 4 of them in the 2nd degree, and only one patient in the 3rd degree obesity category); 13 patients were overweight, with a BMI of between 25 and 30 kg/m^2^, and only 4 other patients were of normal weight. Although, on various occasions, our patients had elevated blood pressure and/or BBG values, they were not suitably diagnosed with T2DM and MS prior to their COVID-19 infection. All patients in this group had associated MS, with a number of defining factors of between 4 and 6, and an average of 5 criteria. During the acute SARS-CoV-2 infection stage, 31 patients suffered a pulmonary injury, affecting around 5 to 40% of their lung parenchyma, with a median of 15% [15–30], thus indicating more severe COVID-19 forms (14 subjects had moderate forms, 32 had mild forms). Consequently, most of them reported multiple persisting symptoms, the average number being of 6 [3.75–7], and also had higher PCFS levels, of 2 [1–3]. As expected, they had significantly higher values of laboratory parameters indicating metabolic dysfunctions than group III (*p* < 0.0001), while the differences, when compared with the data registered for group II, were significant for LDL-cholesterol and eGFR and lipid accumulation product (LAP), especially for the BBG and triglyceride-glucose (TyG) index (*p* < 0.0001), but not for the visceral adiposity index (VAI) (see Table 2).

Regarding the presence of altered TTE parameters, when we performed a comprehensive echocardiographic assessment, we identified several patterns of cardiac abnormalities, even though these patients were not diagnosed with cardiac dysfunctions during the acute COVID-19 infection phase (based on an abbreviated TTE exam).

Although all patients from group I had LVEF values above 50% and their MAPSE was not lower than 10, by using strain techniques, we identified 27 patients with borderline LV-GLS values (from −18 to −19) and an LVEF below 60%. An RVD was identified in 16 patients, with 9 patients also showing slightly elevated PAPs. When referring to the presence of type 1 DD, this was identified in 19 subjects, with type 2 DD in 9 cases, and type 3 DD seen in 3 patients. A thickened pericardium, of between 3 and 4 mm was evidenced in 12 subjects, with one patient also showing a small amount of pericardial effusion (4.8 mm).

Group II included 66 patients (29 men and 37 women), with a mean age of 51.07 ± 4.77 years, all diagnosed with MS, but without T2DM. Their median BMI was of 29.48 [27.49–31.32]. Of them, 30 had obesity (27 patients of a 1st degree, 3 patients of a 2nd degree), 29 were overweight, and 7 had a normal weight. Although none of them had T2DM, all were considered to have MS, as defined by at least 3 factors, with a median value of 4 [4–5]. During the acute phase of COVID-19, 42 patients had sustained pulmonary injuries, with a median value of 10.5% [0–30], which would explain why only 20 subjects suffered from moderate forms of the disease while the remaining had mild forms. At the first presentation, they reported between 2 and 9 persisting symptoms, with an average of 5 [3–7], and a median PCFS scale value of 2 [1–3]. All their laboratory test results, TyG index, VAI, and LAP were significantly higher than those reported in group III (*p* < 0.0001) (as seen in Table 2).

In terms of cardiac abnormalities, as identified by TTE, 33 patients had borderline LVF, certified by LV-GLS (−18 and −19), with 32 of them also showing LVEF values lower than 60%. Regarding the pattern of RVD, 14 patients had a reduced RV-GLS, while 11 also had elevated PAPs. The DD pattern was identified in 37 of patients (of whom, 20 patients had a type 1, 15 patients type 2, while 2 patients had type 3 DD). A thickened pericardium was detected in 12 patients, while one patient also showed a slight pericardial effusion.

Group III included 91 younger individuals, with ages ranging from 26 to 55 (an average age of 41.67 ± 7.44 years), of which 37 were men and 54 women. Although none of them had T2DM, MS, or obesity, their median BMI was of 24.38 [22.56–26.8] kg/m^2^, thus 30 could be considered overweight, while 66 of them had at least one element that is included in the definition of MS; only 25 subjects were in the heathy ranges. Throughout the acute phases of the SARS-CoV-2 infection, the majority suffered mild forms of illness; 15 patients had moderate and 15 had mild lung injuries, so that the median value of the pulmonary impairment was 0% [0–6]. Generally, they had less complaints, reported between 2 and 8 persisting symptoms, with a median of 3 [3–6], and had lower PCFS levels—namely an average of 1 [1–3]. Although more than half of them had at least one or two factors that define MS (mostly, lower HDL-cholesterol levels), their median laboratory values were within normal ranges (as seen in Table 2).

In this subset of patients, significantly fewer cardiac abnormalities were assessed by TTE. For 21 subjects, we evidenced borderline LVF, with LV-GLS values of −18 and −19; 20 of them had an LVEF under 60%. Reduced RV-GLS values were seen in 6 subjects, with 3 of them also having slightly increased PAPs. A DD was determined in 20 patients (type 1 in 14 cases, and a type 2 in 6 subjects). Eight patients had slightly thickened pericardia, while one of them had a slight pericardial effusion.

When analyzing the existence of statistical correlations between the main TTE patterns identified in our patients and several clinical and laboratory parameters, we noticed that the LV-GLS was moderately, but statistically, significantly correlated with the patients’ age, inflammatory markers (namely, CRP, PCFS levels), the number of elements defining MS (TyG, LAP, BMI), and the severity of lung injuries (*p* ˂ 0.0001) (Table 4). RV-GLS was strongly correlated with acute infection severity, namely with pulmonary damage, as assessed on the CCT scan, and with CRP levels (*p* ˂ 0.0001); moderate but statistically meaningful correlations appeared regarding the patients’ age, days since diagnosis, PCFS levels, and the number of factors defining MS, TyG, and LAP (*p* ˂ 0.0001). The E/e’ ratio was strongly correlated with the intensity of inflammation, as expressed by the initial CRP value, and only moderately—but significantly—with the PCFS levels, the severity of the pulmonary injury and time since diagnosis, the number of elements defining MS, the patient’s age, as well as their LAP, TyG, VAI, and BMI (*p* ˂ 0.0001) values. Pericardial thickness was moderately, but significantly, correlated with the severity of the acute infection, as expressed by the degree of pulmonary damage, and the initial CRP levels, and also with the number of days elapsed from initial diagnosis, and PCFS levels (*p* ˂ 0.001) (as seen in Table 4). 

**Figure 2:**
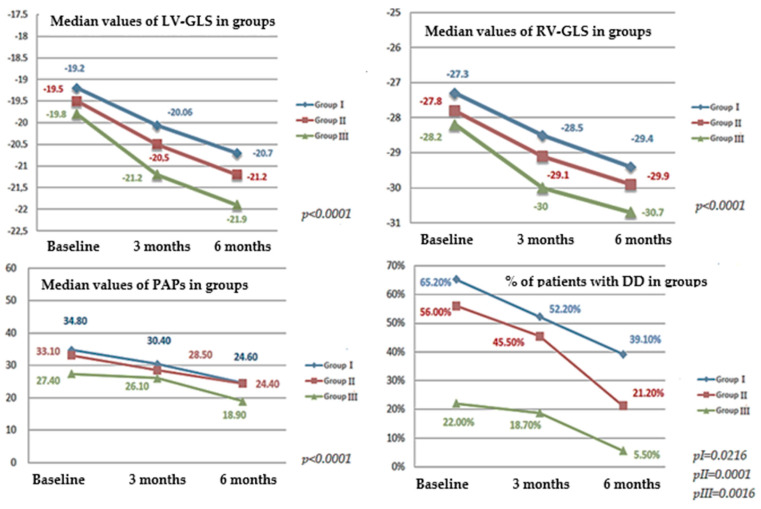
The 6 months evolution of principal cardiac abnormalities assessed by TTE: LV-GLS, RV-GLS, PAPs and DD. Legend: LV-GLS—left ventricular global longitudinal strain; RV-GLS-right ventricular global longitudinal strain; PAPs—systolic pressure in the pulmonary artery; DD—summation of factors: E/A and E/e’ ratio, LAVI and TRV.

Regarding the development of these abnormalities, as assessed by TTE, a meaningful progress could be noted in all study groups (see Figure 2). Thus, in group I, only eight subjects still had borderline LV-GLS at 3 months, while at 6 months these values had normalized; one patient had pathological RV-GLD, while seven had slightly elevated PAPs, however, the RVF normalized at 6 months, with two subjects still showing PAPs borderline values (*p* < 0.0001). Concerning the evolution of the DD, this was less favorable at 3 months.

Overall, 24 patients still showed a DD pattern (14 patients of type 1, 8 patients of type 2, and 2 patients of type 3), while at 6 months, a DD was identified in 18 patients (in 10 cases, there was a type 1, in 7 cases, a type 2, while in 1 case, there still was a type 3). It is worth mentioning that its decline was not statistically significant (*p* = 0.0216) (as seen in Figure 1). For group II, there was a similar evolution. The LVF appeared to gradually recover, so that at 3 months, only four patients still had borderline values of LV-GLS that normalized by the 6-month evaluation (*p* < 0.0001). RVD improved significantly, so that after 3 months, only one patient still had pathological RV-GLS values, while two patients had slightly elevated PAPs, which disappeared after 6 months (*p* < 0.0001). Regarding the DD evolution, this persisted at 3 months for 30 patients (21 of whom had a type 1, 8 a type 2, and 1 a type 3); at the 6-month evaluation, there still remained 14 subjects with a notable DD (7 cases with a type 1, 6 patients with a type 2, and only 1 with a type 3). For group III, the 3-month evaluation showed that only one subject still had borderline LV-GLS values, which normalized at 6 months; the RVD and sPAP appeared to be in normal ranges already after 3 months (*p* < 0.0001), however, DD still was notable at 3 months for 17 patients (14 of whom showed a type 1 DD, while 3 patients showed a type 2 DD); at the 6-month evaluation, 5 patients still suffered from various DD (4 patients had a type 1, while 1 had a type 2 DD, *p* = 0.0016). 

In order to identify the independent predictors that could influence the initial and the six months’ values of LV-GLS, RV-GLS, PAPs, and DD frequency, we used the multivariate direct regression examination method for constructing regression models centered on the forward stepwise technique; the Akaike information criteria (AIC) was used for the selection of the most appropriate model. We excluded data regarding age, BMI, or gender from this analysis, considering that previous studies have already proven that they are strong negative predictors of the aforementioned parameters; therefore, they were considered confounding factors. Consequently, the following parameters were tested in the multivariate regression analysis: CRP, TyG index, BBG, the number of elements defining MS, LAP, and VAI. The independent predictors associated both with the initial and the six months’ values of LV-GLS, RV-GLS, PAPs, and DD frequency are summarized in Table 5.

As visible in Table 5, from all factors included in our regression models, the highest statistical significance for both the initial and the values at 6 months for LV-GLS, RV—GLS, PAPs, and DD was found for the initial level of inflammation (as expressed by CRP serum concentrations), and especially for the TyG index levels (*p* ˂ 0.0001).

## 4. Discussion

In individuals with metabolic dysfunctions, such as obesity, MS, and T2DM, a worse evolution of COVID-19, associated with increased morbidity due to severe complications—frequently requiring ICU admission—and a higher mortality rate, has been discussed in multiple studies, as well as some sizable meta-analyses, ever since the beginning of 2020 [30,31,32,33]. Initially, obesity was considered as an objective health risk-factor for higher morbidity and mortality levels, with the risk, apparently, proportionally increasing with BMI [5]. Thus, it is worth noting that individuals with obesity frequently have associated MS, or even T2DM, which further increases their health risk, favoring the development of various respiratory, cardiovascular, and multi-systemic complications during the course of COVID-19 [5,23,34]. Moreover, it should be highlighted that for this patient category, the inappropriate cardio-metabolic risk profile renders them susceptible toward a delayed/deficient restoration of the immune homeostasis, with the persistence of exaggerated inflammatory processes, responsible for the development of post-acute as well as long COVID-19 conditions [5,28].

The importance of quantifying the increased risk profile determined by these metabolic dysfunctions has become evident and, since IR is their common pathophysiological hallmark, its fast and precocious assessment represents an important issue. Because the hyperinsulinaemia-euglycemia clamp technique, considered the golden standard for the quantitative measurement of IR, is time-consuming and costly, the TyG index has been accepted as an alternative for determining IR [35,36]. Moreover, during the recent pandemic, in some notable reports, a bidirectional relationship between the SARS-CoV-2 infection effects and IR has been evidenced, since this disease appears to favor the IR and β-cell damage due to the release of IL-1β and TNF-α [37,38]. The study of Chang et al. demonstrated a significant association between the TyG index, as determined before COVID-19, and an elevated risk for severe complications during the acute infection [39]. In the same vein, some indexes such as the LAP and VAI—characterizing the abdominal obesity phenotype, which is associated with an impaired risk profile—were considered indicators for a worse COVID-19 outcome [33,40,41]. Starting from these observations, in our study, by analyzing the relationship between these indexes and the TTE parameters characterizing LVF, RVD, and DD, we evidenced statistically significant correlations (*p* < 0.0001) for all of them, but especially for the TyG index, the number of elements defining MS, the level of inflammation (as expressed by the initial CRP values), and the post-acute COVID-19 condition gravity (as quantified by the PCFS scale). As expected, individuals with T2DM and/or MS in our study showed higher levels of these indexes when compared with controls (*p* < 0.0001). According to recent literature studies, as well as concordant with our data, an inappropriate cardio-metabolic risk profile and the IR (as quantified by the TyG index) would predict a worse outcome, with a higher prevalence of cardiovascular complications and an extended recovery period due to the various post-COVID-19 syndrome implications [4,23,33,36]. 

Our research is based on the assumption that even in previously apparently healthy individuals with an inappropriate cardio-metabolic risk profile, undiagnosed(neither before, or during the acute phase of infection) with a significant cardiovascular pathology at a routine TTE, some subtle cardiac deviations could exist, identifiable only in a comprehensive TTE assessment, or, even more accurately, by more sophisticated imaging techniques, abnormalities that could favor the onset of the post-COVID-19 condition [42]. There is a general consensus attesting the wide range of cardiac alterations, identified by TTE, during the infection with the SARS-CoV-2 virus [9,12,43]; their persistence and evolution during the recovery phase are largely debated in current medical literature [28,44]. By means of TTE, in our study, we have managed to identify four main patterns of cardiac alterations. Even in subjects who were classified as having a TTE exam “within normal limits”, we frequently evidenced “borderline” values of the parameters characterizing LVF, RVD, PH, and DD, specifically, in almost half of those with T2DM, MS, and/or obesity. The novelty of our manuscript is that these individuals who are overweight or have grade 1 obesity, and are considered by their GP and by themselves as “apparently healthy”, frequently also have insulin-resistance and can easily fulfil three of the criteria defining MS. This high-risk metabolic profile is often overlooked, and in case of a COVID-19 infection, these patients are prone to develop cardiovascular alterations and post-COVID-19 syndrome. Fortunately, these cardiovascular abnormalities appeared to have been alleviated over time, so that at the 6-month follow-up, the majority of our subjects had predominantly normal values, except most of those with an identifiable DD, where some abnormalities tended to persist longer, raising the suspicion that, due to enduring inflammation, some interstitial fibrotic changes could have occurred in the myocardium, inducing progressive remodeling and stiffening, with an altered relaxation of the cardiac muscle [27,44,45].

Our study’s chief limitation emerges from an unavailability of a detailed TTE exam performed before and during the SARS-CoV-2 virus infection. Although we only selected individuals with a previous TTE examination, we also accepted succinct formulations, such as “within normal limits”, “incipient LVH”, but in most cases, precise measurements of LVMI, LV-GLS, RV-GLS, E/e’ ratio, LAVI, TRV, and sPAP were missing. Consequently, we cannot affirm with the utmost certainty that the subtle TTE abnormalities that we have identified in our study population, such as borderline LVF, mild RVD and/or PH, increased LAVI, or even DD, did not precede the infection, worsening during the acute phase of illness or even during the recovery phase, which appears all the more likely, seeing as a high percent of the individuals included in our study suffered from T2DM, MS, and/or were obese or overweight.

## 5. Conclusions

For people suffering from diabetes mellitus and/or metabolic syndrome—even for those considered apparently healthy before the infection with the SARS-CoV-2 virus—there is a higher probability to develop a post-COVID-19 syndrome, requiring a longer recovery period, at least partially explained by the existence of subtle cardiac abnormalities that can be evidenced by a comprehensive TTE exam. Therefore, patients that belong to an increased cardio-metabolic risk profile category would benefit from also being evaluated by a specialist cardiologist, including having a comprehensive echocardiography routinely offered, in addition to the usual post-COVID-19 assessment, in order to adequately evaluate and address, in a timely manner, all potential serious health consequences.

## Figures and Tables

**Table 1:** Patients’ clinical characteristics.

	Age	BMI	WC		SBP	DBP	HR	No. of Symptoms	PCFS	Days Since First PCR	Initial Lung Injury on CCT Scan
			M	W							
Group I—46 patients with T2DM and MS − 16 men − 30 women − 29 obese − 13 overweight	52 ± 3.54	30.96 [29.22–32.86]	108 [103.25–112]	94 [90–97.5]	130 [120–131]	80 [70–85]	75 [75–80]	6 [3.75–7]	2 [1–3]	56 [56–64.75]	15 [15–30]
Group II—66 patients with MS, but without T2DM − 29 men − 37 women − 30 obese − 29 overweight − 7 normal weight	51.07 ± 4.77	29.48 [27.49–31.32]	105 [103–109.5]	89 [88–93.5]	130 [120–140]	80 [70–90]	75 [75–80]	5 [3–7]	2 [1–3]	63 [56–70]	10.5 [0–30]
Group III—91 controls without T2DM and MS − 37 men − 54 women − 30 overweight − 61 normal weight	41.67 ± 7.44	24.38 [22.56–26.8]	98 [94–100]	79 [70–85]	120 [100–120]	70 [60–70]	80 [75–85]	0 [0–3]	1 [1–3]	63 [63–70]	0 [0–6]
Statistical significance											
I/II	0.2636	0.018	0.176		0.537	0.647	0.256	0.947	0.2018	0.064	0.8349
I/III	<0.00001	<0.0001	<0.0001		<0.0001	<0.0001	0.014	0.005	<0.0001	0.0001	0.0002
II/III	<0.0001	<0.0001	<0.0001		<0.0001	<0.0001	0.0001	0.001	<0.0001	0.0001	0.0001

Legend: BMI—body mass index; WC—waist circumference; M—men; W—women; SBP—systolic blood pressure; DBP—diastolic blood pressure; HR—heart rate; No.—number; PCFS—post-COVID-19 functional scale; PCR—real-time reverse transcriptase–polymerase chain reaction; CCT scan—chest computed tomography scan; T2DM—type 2 diabetes mellitus; MS—metabolic syndrome.

**Table 2:** Patient laboratory characteristics.

	BBG (mg/dL)	Uric Acid (mg/dL)	LDL-Cholesterol (mg/dL)	HDL-Cholest. (mg/dL)	Triglycerides (mg/dL)	CRP (mg/dL)	eGRF (mL/min)	TyG Index	VAI	LAP
Group I—46 patients with T2DM and MS	114.5 [110–120]	7.4 [7.2–8]	140 [130–150]	32.5 [30–40]	170 [160–190]	30.4 [26.28–38.9]	100 [98–105]	4.94 [4.88–5]	3.47 [2.53–4.42]	75.02 [61.4 5–87.35]
Group II—66 patients without T2DM, but with MS	100 [99–100.25]	7.35 [7.2–7.6]	130 [120–141.25]	30 [30–35.75]	162.5 [160–171.25]	30.11 [24–32.61]	104 [100–110]	4.86 [4.83 –4.89]	3.6 [3.05–4.42]	75 [56–191.11]
Group III—91 controls without T2DM and MS	90 [89–95]	6.4 [6–6.8]	100 [90–120]	45 [40–50]	140 [130–145]	26.23 [5.67–40.67]	120 [110–125]	4.72 [4.68–4.75]	2.12 [1.81 –2.6]	41.1 [28.45–51.42]
Statistical significance										
I/II	*p* < 0.0001	*p* = 0.237	*p* = 0.016	*p* = 0.8	*p* = 0.064	*p* = 0.151	*p* = 0.001	*p* < 0.0001	*p* = 0.574	*p* = 0.039
I/III	*p* < 0.0001	*p* < 0.0001	*p* < 0.0001	*p* < 0.0001	*p* < 0.0001	*p* = 0.0001	*p* < 0.0001	*p* < 0.0001	*p* < 0.0001	*p* < 0.0001
II/III	*p* < 0.0001	*p* < 0.0001	*p* < 0.0001	*p* < 0.0001	*p* < 0.0001	*p* = 0.0021	*p* < 0.0001	*p* < 0.0001	*p* < 0.0001	*p* < 0.0001

Legend: BBG—basal blood glucose; LDL—low density lipoprotein; HDL—high density lipoprotein; CRP—C-reactive protein; TyG—triglyceride-glucose index; VAI—visceral adiposity index, LAP—lipid accumulation product; T2DM—diabetes mellitus; MS—metabolic syndrome.

**Table 3:** Patient echocardiographic parameters.

Parameter	Group I—Patients with DM and MS *n* = 46	Group II—Patients with MS, without DM *n* = 66	Group III—Controls without DM and MS *n* = 91	I/II	I/III	II/III
LVMI	98.78 [93.6–110.6]	98.5 [90.3–111.15]	87.74 [70.45–97.45]	0.797	<0.0001	<0.0001
LAVI	28.42 [21.36–34]	23.40 [18.47–31.81]	15.76 [13.32–21.34]	0.059	<0.0001	<0.0001
PT	2.2 [1.5–3.14]	2.2 [1.5–2.8]	1.9 [1.5–2.5]	0.699	0.053	0.104
LVEF	55 [50–60]	55 [50–58]	60 [55–65]	0.995	<0.0001	<0.0001
MAPSE	14 [11–15]	14 [12–16]	17 [15–18]	0.181	<0.0001	<0.0001
LV-GLS	−19 [−21–−18]	−19.5 [−20–−19]	−21 [−22–−20]	0.524	<0.0001	<0.0001
TAPSE	20 [18–22]	20 [18–22]	24 [20–26]	0.726	<0.0001	<0.0001
FAC	35.86 [35–37.89]	36.57 [35.46–37.69]	37.87 [35.8–39]	0.222	0.0004	0.0016
RV-GLS	−28 [−30–−27]	−29 [−30–−28]	−31 [−33–−29]	0.278	<0.0001	<0.0001
TRV	2.7 [2.5–2.73]	2.67 [2.49–2.7]	2.51 [2–2.7]	0.291	<0.0001	<0.0001
PAPs	34.16 [31–34.8]	33.51 [29.85–34.59]	30.20 [21–34.16]	0.291	<0.0001	0.0002
E/A	0.97 [0.8–1.29]	1.02 [0.76–1.26]	1.11 [0.92–1.34]	0.095	0.206	0.1612
E/e’	14.08 [11.55–14.32]	13.50 [11.67–14.24]	11.92 [9.87–13]	0.503	<0.0001	<0.0001

Legend: LVMI—left ventricular mass index; LAVI—left atrial volume index; PT—pericardial thickness; LVEF—left ventricular ejection fraction; MAPSE—mitral annular plane systolic excursion; LV-GLS—left ventricular global longitudinal strain; TAPSE—tricuspid annular plane systolic excursion; FAC—fractional area change; RV—GLS-right ventricular global longitudinal strain; TRV—peak tricuspid regurgitation velocity; PAPs—systolic pressure in the pulmonary artery; E/A—peak early mitral inflow velocity (E) to late diastolic velocities (A) in pulsed Doppler; E/e’—early mitral inflow diastolic velocity (E) to average velocity at the level of the mitral ring in pulsed tissue Doppler’.

**Table 4:** Associations between the TTE parameters and several clinical and biological data.

Parameter	LV-GLS	RV-GLS	E/e’	PT
Age	r = 0.45, *p* < 0.0001 95%CI [0.334–0.554]	r = 0.62, *p* < 0.0001 95%CI [0.538–0.706]	r = 0.45, *p* < 0.0001 95%CI [0.339–0.558]	r = 0.15, *p* = 0.032 95%CI [0.0126–0.281]
BMI	r = 0.36, *p* < 0.0001 95%CI [0.236–0.476]	r = 0.33, *p* < 0.0001 95%CI [0.202–0.448]]	r = 0.36, *p* < 0.0001 95%CI [0.242–0.480]	r = 0.054, *p* = 0.438 95%CI [−0.083–0.19]
Lung injury	r = 0.35, *p* < 0.0001 95%CI [0.231–0.472]	r = 0.71, *p* < 0.0001 95%CI [0.644–0.778]	r = 0.62, *p* < 0.0001 95%CI [0.530–0.700]	r = 0.51, *p* < 0.0001 95%CI [0.410–0.613]
Days since dg.	r = −0.28, *p* < 0.0001 95%CI [−0.408–−0.155]	r = −0.61, *p* < 0.0001 95%CI [−0.691–−0.517]	r = −0.48, *p* < 0.0001 95%CI [−0.584–−0.372]	r = −0.61, *p* < 0.0001 95%CI [−0.691–−0.518]
PCFS	r = 0.51, *p* < 0.0001 95%CI [0.409–0.611]	r = 0.68, *p* < 0.0001 95%CI [0.599–0.748]	r = 0.63, *p* < 0.0001 95%CI [0.544–0.710]]	r = 0.44, *p* < 0.001 95%CI [0.332–0.553]
No. of MS elements	r = 0.42, *p* < 0.0001 95%CI [0.307–0.533]	r = 0.59, *p* < 0.0001 95%CI [0.493–0.674]	r = 0.47, *p* < 0.0001 95%CI [0.355–0.571]	r = 0.22, *p* = 001 95%CI [0.094–0.355]
CRP	r = 0.53, *p* < 0.0001 95%CI [0.431–0.628]	r = 0.74, *p* < 0.0001 95%CI [0.671–0.797]	r = 0.74, *p* < 0.0001 95%CI [0.679–0.802]	r = 0.50, *p* < 0.0001 95%CI [0.391–0.598]
TyG index	r = 0.43, *p* < 0.0001 95%CI [0.391–0.542]	r = 0.53, *p* < 0.0001 95%CI [0.432–0.629]	r = 0.43, *p* < 0.0001 95%CI [0.319–0.542]	r = 0.23, *p* = 0.0008 95%CI [0.099–0.359]
VAI	r = 0.28, *p* < 0.0001 95%CI [0.148–0.403]	r = 0.31, *p* < 0.0001 95%CI [0.192–0.440]	r = 0.39, *p* < 0.0001 95%CI [0.275–0.507]	r = 0.13, *p* = 0.051 95%CI [−0.00082–0.269]
LAP	r = 0.38, *p* < 0.0001 95%CI [0.265–0.499]	r = 0.45, *p* < 0.0001 95%CI [0.343–0.561]	r = 0.44, *p* < 0.0001 95%CI [0.327–0.549]	r = 0.19, *p* = 0.005 95%CI [0.058–0.323]

Legend: LV-GLS—left ventricular global longitudinal strain; RV-GLS—right ventricular global longitudinal strain; E/e’—early mitral inflow diastolic velocity E to average e’ velocity (E/e’) in pulsed tissue Doppler; PT—pericardial thickness; BMI—body mass index; PCFS—post-COVID-19 functional scale; CRP—C-reactive protein; TyG—triglyceride-glucose index; VAI—visceral adiposity index, LAP—lipid accumulation product.

**Table 5:** Multivariate regression analysis used for the identification of objective predictors of LV-GLS, RV-GLS, DD, and PAPs initial values and six months’ values.

Predictors	Baseline			End of Follow Up (6 Months)		
	β	±SE	*p*	β	±SE	*p*
Multivariate linear regression analysis of LVF (LV-GLS)						
CRP (mg/dL)	β = 0.064	±0.009	*p* < 0.0001	NS	-	-
TyG index	β = 3.12	±0.75	*p* = 0.0001	β = 4.25	±0.73	*p* < 0.001
Multivariate linear regression analysis of RVF and PH (LV-GLS, respectively PAPs)						
CRP (mg/dL)	β = 0.136 β = 0.044	±0.010 ±0.005	*p* < 0.0001 *p* < 0.0001	β = 0.039 β = 0.24	±0.009 ±0.092	*p* = 0.0001 *p* = 0.011
TyG index	β = 6.96 β = 3.30	±2.53 ±0.82	*p* = 0.0065 *p* = 0.0001	NS β = 0.018	- ±0.0071	- *p* = 0.009
Multivariate linear regression analysis of DD frequency						
CRP (mg/dL)	β = 0.567	±0.036	*p* < 0.0001	β = 0.023	±0.0044	*p* < 0.0001
TyG index	β = 14.89	±2.97	*p* < 0.0001	β = 1.332	±0.362	*p* = 0.0003

Legend: LVF—left ventricular function; LV-GLS—left ventricular global longitudinal strain; CRP—C-reactive protein; TyG—triglyceride-glucose index; RVF—right ventricular function; PH—pulmonary hypertension; RV—GLS-right ventricular global longitudinal strain; PAPs—systolic pressure in the pulmonary artery; DD—diastolic dysfunction; β—regression coefficient. SE—standard error. *p*—statistical signification.

## Data Availability

Our data are available on Mendeley at https://doi.org/10.17632/scrxnk26gs.2/ accessed on 23 February 2023.
